# Wireless and battery-operatable IoT platform for cost-effective detection of fouling in industrial equipment

**DOI:** 10.1038/s41598-024-64675-4

**Published:** 2024-06-18

**Authors:** Julius Korsimaa, Martin Weber, Petteri Salminen, Joonas Mustonen, Denys Iablonskyi, Edward Hæggström, Arto Klami, Ari Salmi

**Affiliations:** 1 Electronics Research Laboratory, Department of Physics, University of Helsinki, Helsinki, Finland; 2 Department of Computer Science, University of Helsinki, Helsinki, Finland

**Keywords:** Electrical and electronic engineering, Electronic and spintronic devices, Applied physics, Acoustics

## Abstract

We present a novel internet of things (IoT) sensing platform that uses helical propagation paths of ultrasonic guided waves (UGWs) for structural health monitoring. This wireless sensor network comprises multiple identical sensor units that communicate with a host PC. The units have dedicated hardware to both generate and receive ultrasonic signals, as well as RF signals for use in triggering the sensors. The system was developed for monitoring and sensing pipelines and similar structures in real-time to facilitate interactive sensing. For accurate sensing with a limited number of arbitrarily scattered sensors, we obtain information from all sensor pairs and analyze helical propagation paths in addition to the commonly used shortest paths. UGWs can propagate long distances along the walls of pipelines, and their propagation velocity depends directly on the thickness of the waveguide, and is affected by energy leakage and mass loading. In this paper, we evaluated the network by utilizing it to detect fouling. The network could be adapted for further ultrasonic measurement tasks, e.g., measuring wall thicknesses or monitoring defects with pulse-echo methods.

## Introduction

Efficient and reliable operation of large-scale industrial equipment reduces operational costs and environmental impact. This goal can be achieved by using IoT sensing systems and predictive maintenance methods^1^. Common industrial equipment includes pipelines, which are prone to the accumulation of unwanted sediments called fouling^2^. Fouling accumulates over time, increasing flow resistance and lowering thermal conductivity in pipes, leading to reduced efficiency. Therefore, access to real-time information on fouling accumulation could benefit predictive maintenance over the life cycle of equipment. In most cases, secondary indicators for fouling detection are used^3^, as it is challenging to monitor fouling inside pipes directly. Such indicators might be, for example, a decrease in pressure along the equipment. Direct localization of fouling could be beneficial as it gives a more detailed picture of the equipment and further allows the cleaning of the fouled pipe sections without affecting the rest of the system.

Ultrasonic guided waves (UGWs) are used to investigate pipelines over long ranges^4–7^ due to their sensitivity to fouling^8,9^. The wave propagation speed depends on the effective thickness of the pipeline wall, and accumulated fouling has the effect of increasing the effective wall thickness. Furthermore, the fouling layer causes energy leakage from the wall, reducing the amplitude of the guided wave. The ultrasonic signal traveling from the transmitter toward the receiver at a far location will be affected by the cumulative effect of the fouling along that path. As such, aggregate observations from multiple sensors and/or multiple helical propagation paths between sensor pairs are needed to localize the fouling. For constant monitoring of large structures, numerous, permanently attached ultrasonic transducers and data acquisition equipment are needed, which makes this approach economically unfeasible^10,11^.

The traditional localization approach is to use collar-shaped transducer arrays in the pulse-echo mode utilizing longitudinal waves, thus the approximate defect location can be estimated from the time-of-flight measurement of the echo signal. Guided wave tomography (GWT) methods allow for more accurate defect characterization, however, they also require a large number of sensors placed around the defect thus covering only a small area^12–14^. Applying GWT methods to cylindrical structures such as pipelines allows to achieve high defect reconstruction accuracy with a small number of sensors benefiting from higher order helical signal propagation^15–17^. Typical acquisition systems consist of costly modules for signal generation, amplification, and multichannel oscilloscopes, thus limiting the applications to laboratory environments. In addition, arrays are difficult to deploy if surrounding structures restrict access to the pipe.

Wireless IoT sensing systems have several advantages such as reduced size, easy deployment, self-contained sensor node, while providing same performance as traditional sensing platforms^18^. Low-cost wireless solutions found in the literature are limited either to local measurements of, e.g., wall thickness^19,20^, measurements with the direct propagation paths^21^ or impact detection^22^. Such systems are unable to efficiently monitor large structures with sufficient resolution with a reduced number of transducers.

We present a cost-efficient, battery-operated wireless IoT sensor platform designed to utilize the helical propagation paths of UGWs to continuously monitor fouling in industrial equipment. To reduce the number of transducers required to monitor a length of pipe, we evaluate the helical propagation paths, which gives additional information about the structure when compared to just the shortest path between sensors. The individual identical sensors can be placed arbitrarily, which allows their locations to be determined based on the industrial environment rather than classical design. Additionally, the sensor unit is based on standard components to reduce each node’s cost and to ensure compatibility with a variety of ultrasound sensors. These properties enable a scattered network of permanently attached sensors for continuous monitoring. The system was validated in a laboratory setting by demonstrating the localization of a fouling proxy.

The main advantages of our solution are the capability to place sensors arbitrarily, and the reduced number of sensors required to cover sections of equipment.

We build upon our previous work^23^, by redesigning the signal generation and acquisition circuits for improved performance, as well as including a wireless triggering method. We present details on the individual sensor units as well as the network they form. Finally, we demonstrate that data produced by a network of four sensors can be used to localize fouling attached to a steel pipe, and distinguish different fouling locations.

## Methods

Different structures allow the propagation of different kinds of UGWs^24^. For solid plates in a vacuum, the UGWs are called Lamb waves with two different kinds of displacement profiles, symmetric and asymmetric, S- and A-modes, respectively^25^. To ease signal analysis, a suitable combination of excitation frequency and plate thickness (frequency-thickness product) can be selected to generate only the 0th orders of these modes (A0, S0). In the setup used in this study, frequencies below 800 $\hbox {kHz}$ generate only the fundamental modes. In the case of a steel-to-fluid boundary, the A0 mode has a larger out-of-plane displacement compared to the S0 mode^26^. This was verified empirically by comparing the received signal amplitudes and with the open source “Dispersion Calculator” software^27^. Therefore, the energy transfer of the A0 mode is commonly preferable for fouling detection.

The presence of fouling on the walls of a pipe may be observed as attenuation in ultrasonic signals as the fouling layer enables energy leakage from the waveguide to the fouling layer. Well-adhered fouling can also change the apparent thickness of the pipe. UGW propagation depends on the frequency-thickness product of the waveguide, so a change in the apparent thickness of the pipe may change their propagation velocity^28^. If the used excitation energy is low, higher-order modes will not be generated.

Monitoring these changes aids the assessment of the fouling condition in the pipe^8,28–32^. This is possible, as the thickness of the wall is much smaller than the diameter of the pipe. Therefore, the pipe wall is comparable to an infinite plate with a periodic boundary condition^33^, with which there is no difference between the top and bottom surfaces. As the fouling proxy is placed dozens of wavelengths apart from the transducers, an idealized guided wave can be assumed. This simplifies the experimental work as a fouling phantom can be applied onto the outside of the pipe^34^.

In the case of pipe walls, UGWs can travel not only along the shortest, direct path between two points but also helically along the pipe wall (Fig. 2)^5–7^. The helical paths can be classified by their chirality, i.e. whether the path curls clockwise or anti-clockwise when viewed along its central axis, and the order starting with the direct path as 0th order. The practical utility of higher orders is limited by geometrical attenuation and the ability to differentiate between wave packets in the received signal due to overlapping.

A common approach to structural monitoring is to reduce the detection problem to only the direct path between two transducers. As a drawback, this solution requires moving a pair of transducers, or using two arrays of transducers to find defects, which increases manual work and system cost, respectively. To avoid these drawbacks, one can utilize the helical propagation paths to increase the coverage of a transducer pair^23^. With appropriate data analysis and transducer positioning, adequate signal quality can be achieved, and comparative results can be obtained with reduced hardware. By considering helical paths, the sensing becomes less sensitive to the locations of the transducers, as information can be retrieved along the entire circumference of the pipe. One can achieve a resolution capable of localizing fouling with just a few transducers.

**Figure 1:**
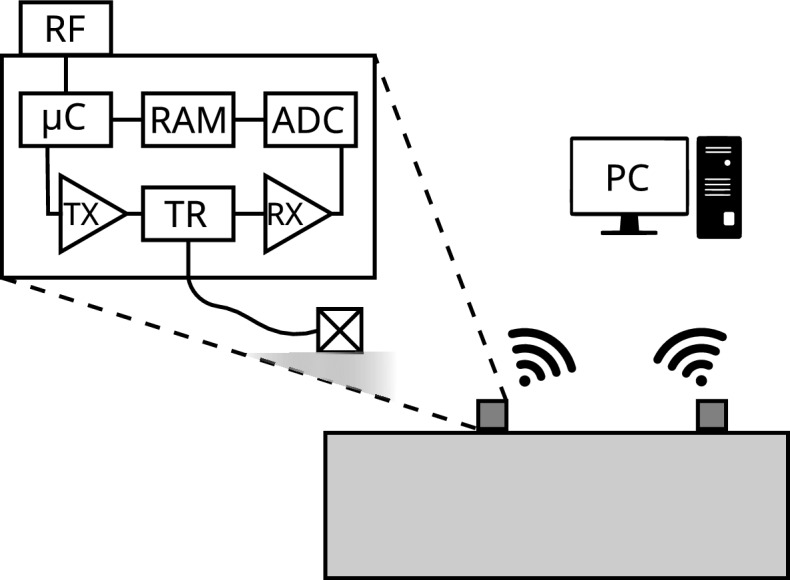
Schematic of the sensor network on a pipe. The network has multiple sensor units connected wirelessly to a host computer. The signal chain of a sensor unit consists of a microcontroller (μC) with additional memory (RAM), TX and RX amplifiers, a TR switch, an ADC, an RF transceiver, and a transducer.

**Figure 2:**
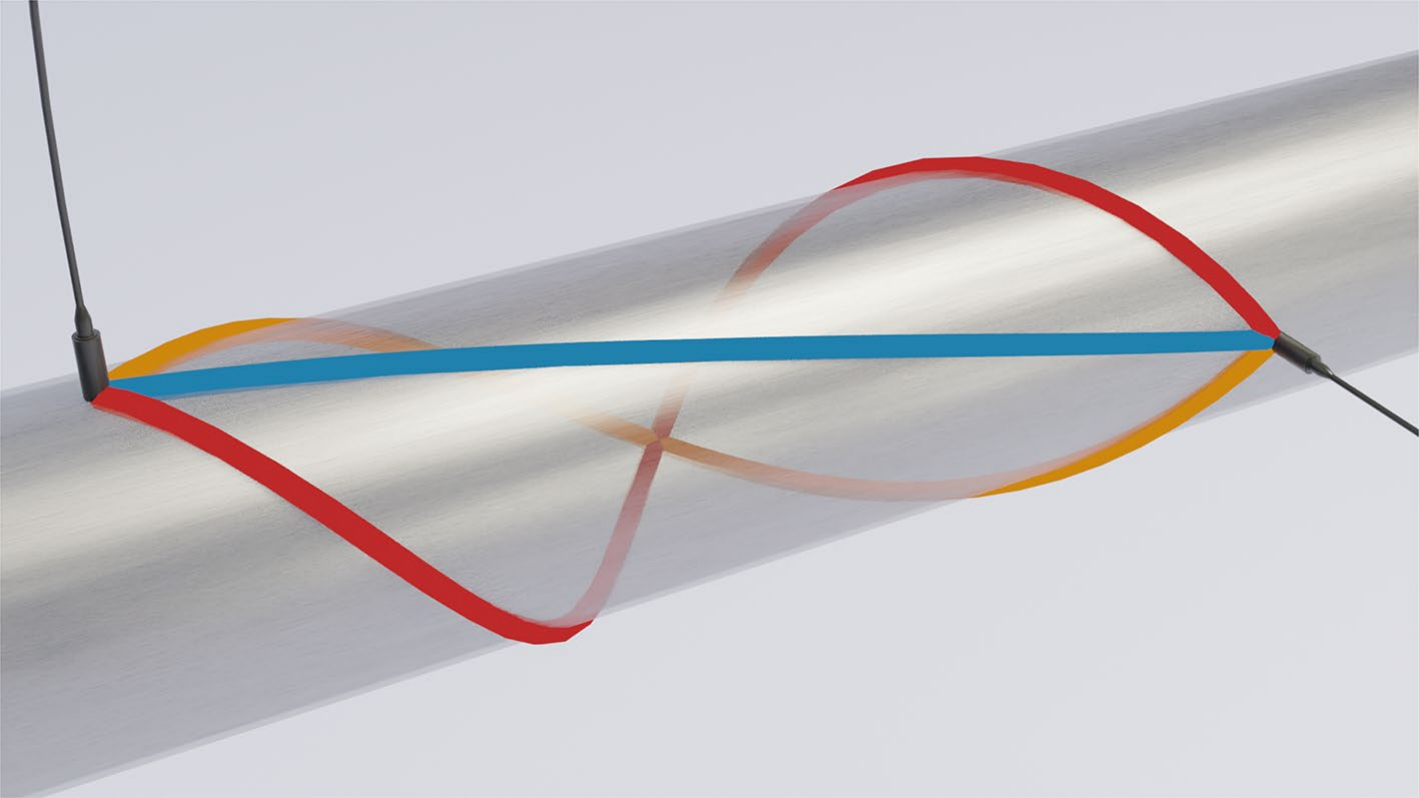
Ultrasonic waves can propagate between two points in a pipe wall in a direct path (blue), or helical paths in either the left-hand (orange) or right-hand (red) direction. Helical paths with higher orders exist and are relevant for the detection of fouling but have been omitted for clarity. Fouling can be detected if any of the paths intersect the fouling.

## Implementation

### Network

Sensor configuration and the measurement protocol are controlled with a host computer running a Python 3 interface. Measurement parameters such as the frequency and length of the actuation signal, and the number of recorded samples can be adjusted based on the properties of the monitored structure. When connected to a host, the sensors assume an idle state where only the microcontroller is powered on, and wait for commands from the host. The host can set the sensors into three states: receiver, transmitter, and sleep.

When acting as a receiver, the sensor initializes the receiving (RX) circuit and waits for a trigger by monitoring the radio frequency (RF) transceiver. When a trigger is received, a set number of samples are stored in the external random-access memory (RAM). After signal reception, the RX circuit is powered down to an intermediate state where only the RAM is enabled. Finally, the recorded data is transferred from external memory to the microcontroller’s internal memory, and the RX circuit is fully powered down as the sensor returns to idle mode. The host can then retrieve the data from the microcontroller with a subsequent read command.

As a transmitter, the sensor enables its transmitting (TX) circuit and sends a message with the RF transceiver. The sensor then waits for a confirmation of the message being sent, which triggers the actuation signal. After sending the ultrasound signal, the sensor returns to idle mode. The trigger signals have a standard deviation of 2.6 ns (100 samples) in the time of arrival when used in a laboratory setting, making the solution suitable for our purposes.

If the host disconnects from the network, the sensors assume a sleep mode, where they periodically search for the host but otherwise conserve power. With an uptime of 2 %, a sensor could operate for a month on a 39 Wh battery at room temperature.

### IoT sensor unit

**Figure 3:**
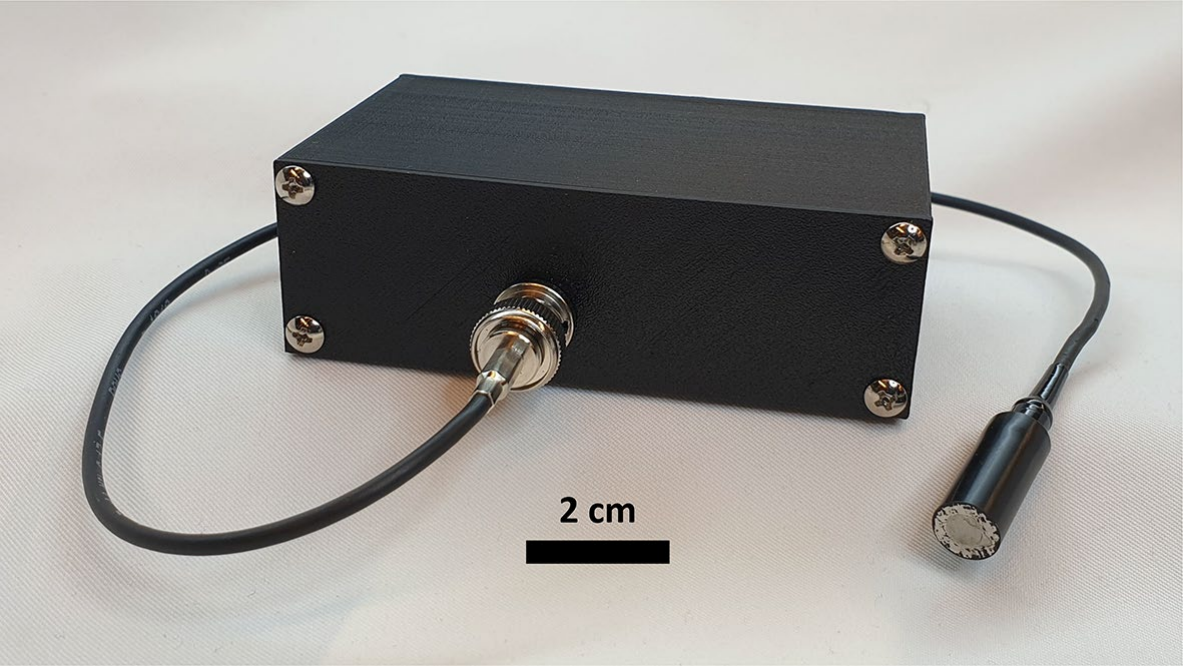
A photograph of a sensor unit with a transducer attached. The transducers consist of a piezo disk ($\varnothing$5 mm) embedded into a POM housing ($\varnothing$10 mm). The transducers are connected to the sensor units with a BNC connector.

The sensor unit (Figs. 1 and 3) is based on the ESP32-WROOM-32E (Espressif, Shanghai, China) microcontroller module, which enables wireless communication (IEEE 802.11n) between a host computer and the sensor unit for configuration and data transfer^23^. To facilitate measurements, the sensor unit has an ultrasound transceiver for transmitting and receiving ultrasonic signals. The TX signal is generated by the digital output of the microcontroller, which drives a single-MOSFET class D amplifier. The amplified signal is used to drive a piezoelectric transducer, which generates ultrasonic pulses.

The received ultrasound produces electrical signals to the order of milli- or microvolts. Therefore, the received electrical signals are amplified by an RX amplifier consisting of two operational amplifiers (LMH6609, Texas Instruments, Dallas, USA), with a configurable voltage gain of up to 54 dB with a − 3 dB bandwidth of 6 MHz. A T/R switch (MD0100, Microchip, Arizona, USA) protects the RX circuit from overvoltage during transmission. The amplified signal is digitized by a dedicated ADC (AD9200, Analog Devices, Wilmington, USA) to external RAM (IS61WV6416DBLL, ISSI, Milpitas, USA) with a sampling frequency of 4 MHz. During signal reception, a predefined number of samples are stored in the memory, pre-processed, and then transmitted wirelessly to the host. The sensors are triggered with a radio frequency (RF) transceiver (DWM1000, Qorvo, Greensboro, USA).

### Transducers and coupling

**Figure 4:**
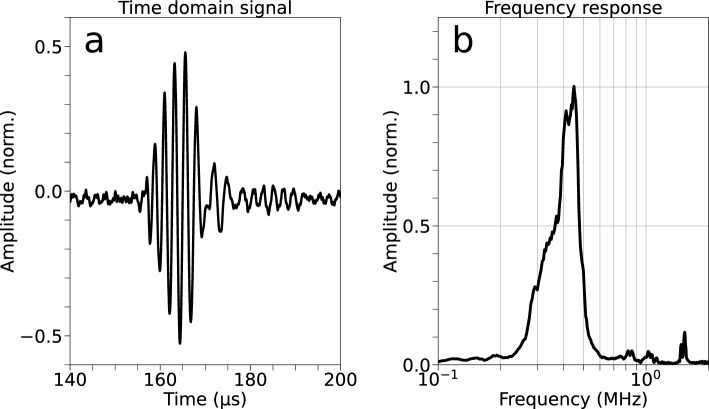
(**a**) The time domain waveform of the through transmission measurement. The waveform is consistent with wide-band transducers. (**b**) The spectrum of the time-domain signal, obtained by Fourier transform, shows a 435 $\hbox {kHz}$ center frequency, with a − 6 $\hbox {dB}$ bandwidth of approximately 366 $\hbox {kHz}$ to 488 $\hbox {kHz}$.

The ultrasonic transducers (Fig. 3) were manufactured to our specifications by Optel (Wrocław, Poland). They consist of a $\varnothing$5 mm piezo disk embedded into a $\varnothing$10 mm polyoxymethylene (POM) housing. The bandwidth (366 kHz to 488 kHz, − 6 dB) of the transducers was determined with a pulse echo measurement on the steel pipe used in the experiments. The time domain signal and its spectrum are shown in Fig. 4.

A coupling medium (Super Adhesive Grease, CRC Industries, Horsham, USA) was used to ensure proper acoustic coupling between the transducers and the pipe wall. The medium was selected due to its good temporal stability. To verify the long-term stability of our coupling method, a seven-month-long series of measurements were conducted in a laboratory environment with fairly constant temperature (21 °C to 24 °C ) and humidity (10 RH to 70 RH). The setup comprised a 3D printed clamp, two transducers (S 12 HB 0,8-3, Karl Deutsch, Wuppertal, Germany), brass delay line (73 mm, $\varnothing$40 mm). For each measurement, a transducer was driven (600 $\hbox {kHz}$, $U_{pp}=$ 10 V, 5 cycles) with a signal generator (AFG31102, Tektronix, Beaverton, USA) and the signal from the other transducer was recorded with an oscilloscope (HDO6104, LeCroy, Chestnut Ridge, USA). Very little change in signal amplitude was observed, and thus the method was used for the sensor network experiments. Long-term stability is crucial for this application, as the transducers should stay attached to the monitored equipment for long periods of time without affecting the measurement.

## Measurements and evaluation

### Fouling experiments

**Figure 5:**
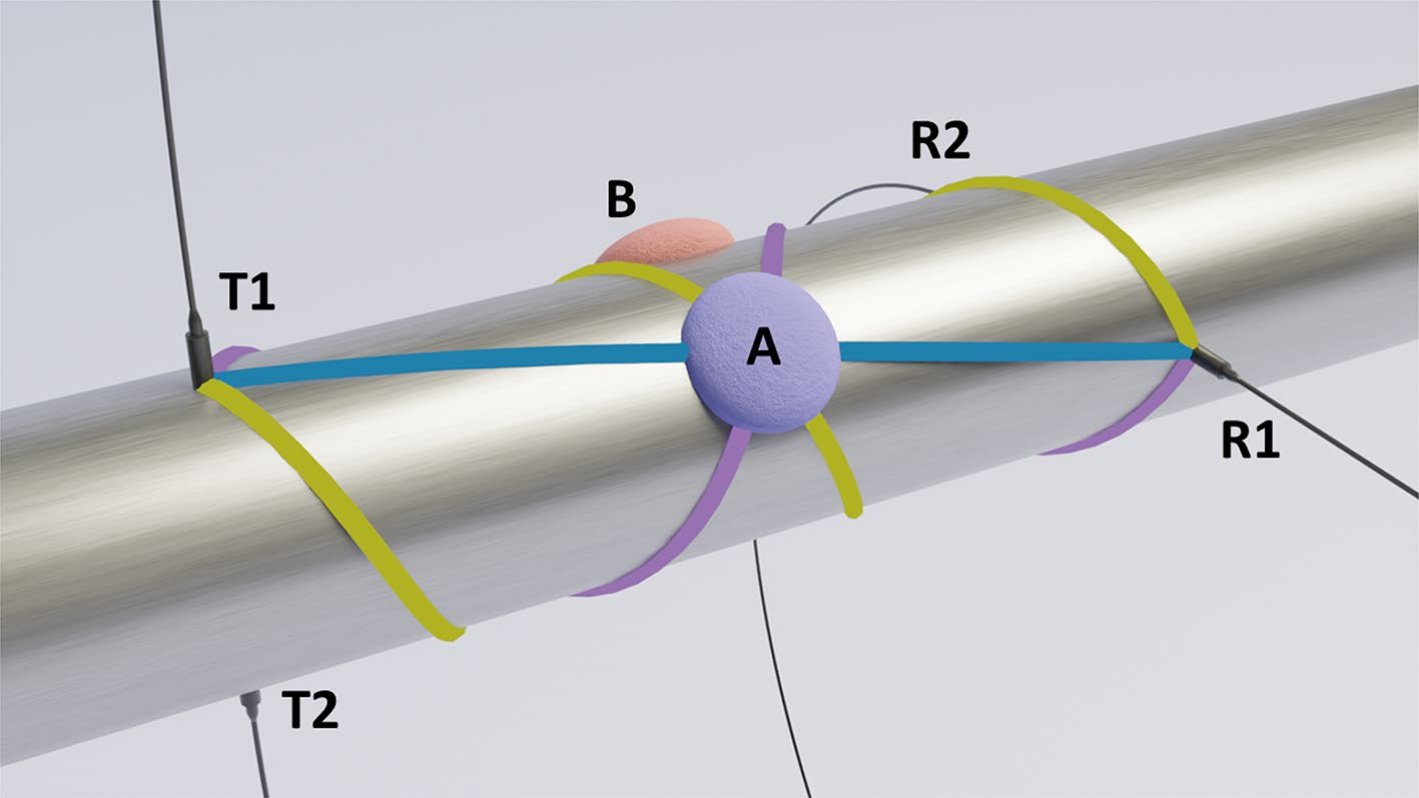
A 3D rendering of the measurement setup. The setup includes four transducers (T1, T2, R1, R2), and two fouling phantom locations: A and B. The location of the fouling phantom depended on the experiment. Helical paths from T1 to R1 intersecting the fouling location are also visualized. The helical paths have been color-coded according to Fig. 6.

Two properties of the network were tested: stability and fouling detection. In both experiments, a measurement set consisted of 10 measurements with the entire network, producing data from all possible sensor pairs of the four-sensor network. The experiments were conducted on a 2.54 m long steel pipe (DIN 11850, material number 1.4404) with an inner diameter of 150 mm and a wall thickness of 2 mm. An air-filled pipe was used to amplify the observed effects of the fouling. Two pairs of transducers were attached to the pipe, positioned to maximize the difference in path lengths between different helical propagation paths for the pipe used. The first pair was attached 25 cm left of the center of the pipe at angles of 0° (T1) and 180° (T2). The second pair was attached 25 cm right of the center at 90° (R1) and 270° (R2) angles (Fig. 5). A fouling phantom was placed at the center of the pipe with polar coordinates of 45° (location A) or 315° (location B) depending on the experiment. As a convention, if the fouling proxy is not placed on the pipe, the pipe is considered ”clean”, whereas the pipe with a fouling proxy attached is ”fouled”.

### Fouling proxy

**Figure 6:**
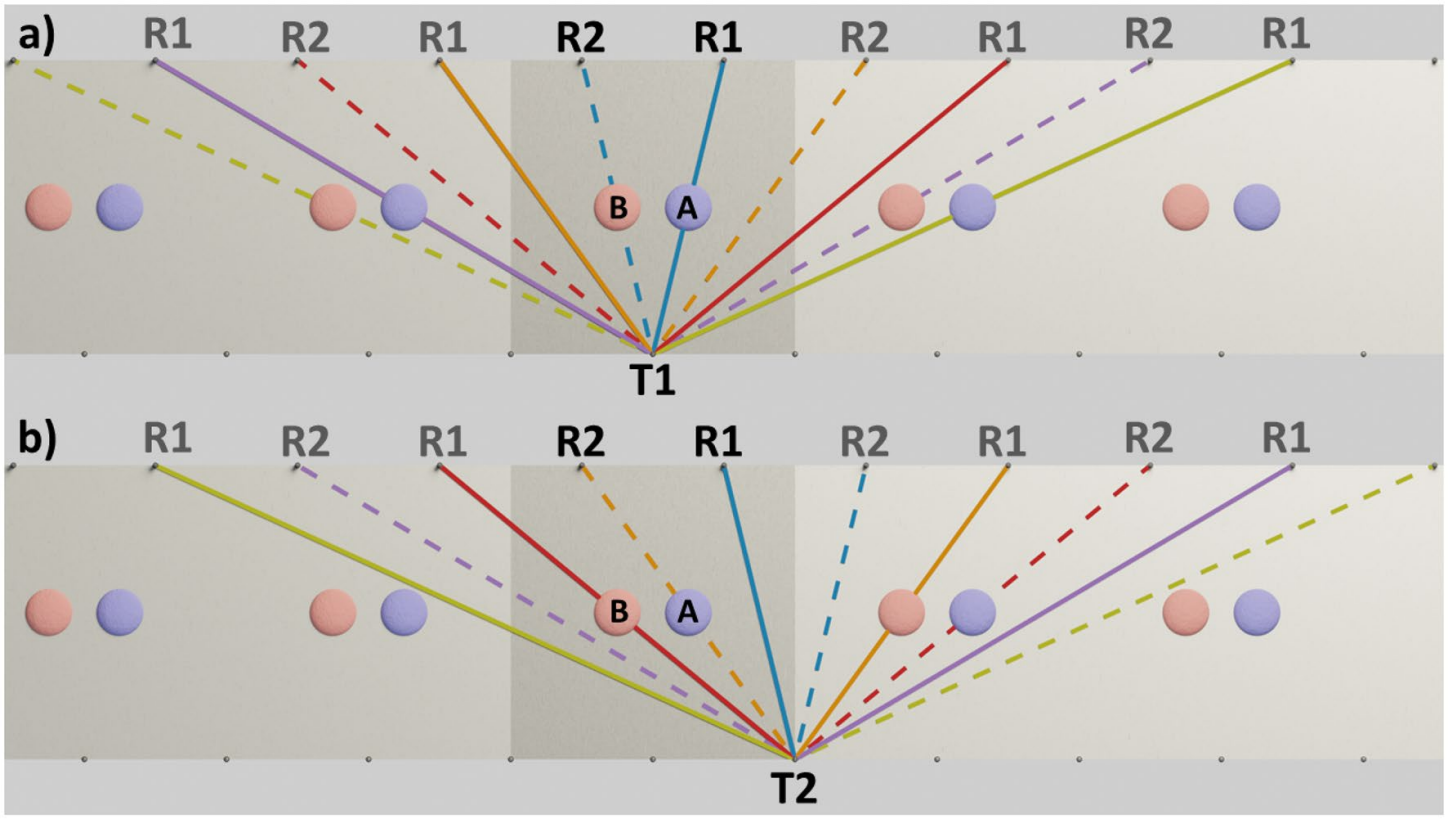
A 2D representation of the measurement setup. Virtual unwrapped pipe surfaces (light gray) have been added alongside the real surface (dark gray) to visualize the periodic nature of the pipe. (**a**) Shows the possible propagation paths for sensor pairs T1-R1 (solid) and T1-R2 (dashed), and whether they intersect with fouling. The paths are, from the shortest to the longest: Blue, orange, red, purple, and yellow. The two fouling locations are marked in blue (A) and red (B). (**b**) Shows the first five propagation paths for transducer pairs T2-R1 (solid) and T2-R2 (dashed). One can see that different paths are affected. The color-coding in this figure will be used throughout the paper.

Adhesive pads (Klebepads, HERMA, Filderstadt, Germany) were used as the fouling phantom, as they were readily available and easy to attach and remove in a controlled manner. The 100 g proxy was spread evenly over a circular area ($\varnothing$8 cm). Two fouling configurations (A, B) were tested on the pipe. The locations were chosen to intersect with distinct direct and helical paths (Fig. 6).

### Evaluation

Stability was tested by comparing a baseline measurement set from a clean pipe to a control measurement set obtained on a clean pipe after all other measurement sets. The purpose of this experiment was to verify that any effect seen in future signals could be reliably attributed to the fouling proxy instead of accidental disturbances in the setup.

To ascertain the effect of fouling five measurement sets were produced. First, a baseline measurement was conducted on a clean pipe, followed by a measurement with the fouling proxy in location A. After removing the proxy, the clean pipe was measured again. Finally, the proxy was placed in location B, followed by a final measurement of the clean pipe. The goal of this experiment was to show that the difference between each measurement set on the clean pipe was negligible, while the effect caused by fouling in both cases of a fouled pipe was detectable. Additionally, the fouling cases should be distinguishable.

## Results

To ease the analysis of data some simplifications can be made. Due to reciprocity, the transducers can be treated as transmitter-receiver pairs, and only one signal direction needs to be recorded. In addition, sensor pairs T1-T2 and R1-R2 are ignored as they are positioned close to each other and their signals do not cover the section of the pipe under investigation. This leaves four TX-RX pairs of interest (T1-R1, T1-R2, T2-R1, T2-R2).

### Stability of setup and effect of fouling

Let us consider the sensor pair T1-R1 for the following analyses (Fig. 6a, solid).

We analyzed the amplitude to compare between the measurements. Energy leakage into the fouling layer reduces the signal amplitude, which makes the change simple to analyze as only binary results of the fouling effect were needed. The comparison points were manually picked from the first baseline signal, at the maxima of the first five wave packets of the A0 mode. The wave packets were identified by calculating the expected time of arrival from the speed of sound in the waveguide and the distance covered by the wave. The same points in time were used for all measurements. The signals were cross-correlated to ensure alignment.

The data produced by the stability experiment shows that the difference of the mean amplitude maxima from the clean and control measurements are smaller than 2%. They also have comparable 2$\sigma$ confidence intervals. The result shows that the setup is robust enough that the amplitude changes caused by the fouling proxy application process are noticeably smaller than the effect of the fouling proxy itself.

The effect of fouling was evaluated from the apparent amplitude drop at the previously defined points in the signals, using data from the same sensor pair. Each signal was sampled 10 times. As the difference between all measurements on a clean pipe was negligible, the effect of fouling was compared against the first baseline measurement. In fouling case A, apparent drops of $(47.9\pm 0.5)\%$, $(53.1\pm 0.7)\%$, and $(49.8\pm 1.4)\%$ in amplitude are observed in wave packets 0, 3, and 4, while wave packets 1 and 2 show a decrease of $(0.8\pm 0.4)\%$ or less. In fouling case B, there is a difference of under $2\%$ in the first four amplitude maxima, as none of the propagation paths intersect the fouling proxy. The fifth maximum experienced a $(12.7\pm 1.1)\%$ decrease, presumably due to, e.g., scattering from the nearby fouling proxy.

**Figure 7:**
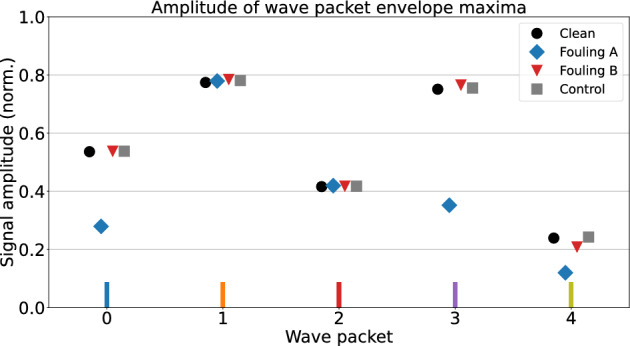
Recorded envelope amplitude maxima of the first five wave packets of the recorded time-domain signals, when T1 is transmitting and R1 is receiving. The baseline and control measurements on a clean pipe produce similar results, as expected. With fouling configuration A, the paths affected by fouling (0,3,4) show noticeably decreased amplitudes, while the other paths (1,2) are not affected by fouling. With fouling configuration B, no effect from fouling is detected on the first four wave packets. Both results are corroborated by the geometry of the setup (Fig. 6). The wave packets have been marked with bars (bottom) according to Fig. 6.

Fig. 7 shows the signal envelope amplitudes at the expected arrival times of the corresponding wave packets. Comparing the data from the clean measurement to the control measurements shows little change in both amplitude and standard deviation, whereas fouling configuration A shows a great decrease in amplitude in the aforementioned wave packets. As such, the changes in amplitude are likely to be caused by the fouling phantom. Conversely, fouling case B has no effect on the signal and cannot be distinguished from the first two measurements, apart from the last wave packet.

### Fouling localization

Geometrical consideration of the propagation paths between sensors T1 and R1 shows that of the two fouling locations only fouling in location A can be detected. To detect fouling in location B, data from other sensors needs to be included.

**Figure 8:**
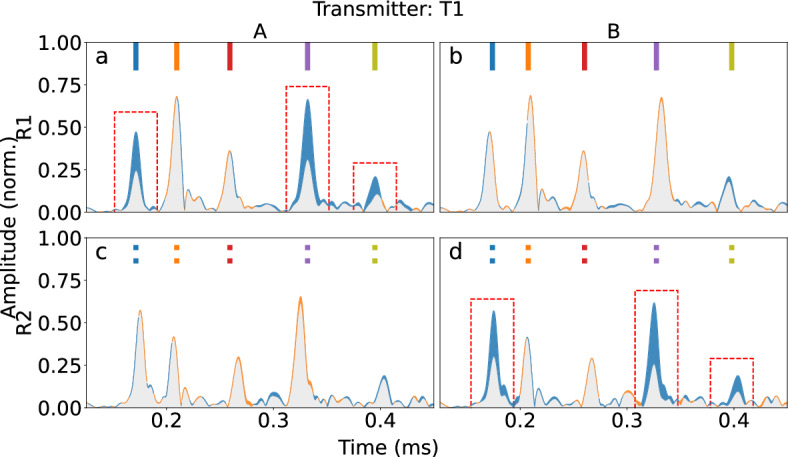
Envelopes of the time-domain signals where T1 is transmitting. Blue areas of the envelopes mark a decrease in amplitude compared to the baseline, whereas orange areas mark an increase in amplitude. The column titles show the fouling location (A, B) and the rows denote the receiving unit (R1, R2). The wave packets have been marked with bars following the color-coding of Fig. 6. The drops in amplitude (boxed) seen in this figure correspond to paths intersecting fouling in Fig. 6a.

Adding a second receiver (R2) to the network produces a 2D matrix (Fig. 8) from the signals received by R1 and R2 as T1 actuates in fouling cases A and B. Each subplot shows the difference between mean signal envelopes of a baseline signal and a signal from a fouled pipe. Increases or decreases in signal amplitude are colored in orange and blue, respectively.

As before, helical paths of 0, 3 and 4 between T1 and R1 intersect with fouling A (Fig. 8a), causing a decrease in amplitude between the two signal envelopes. Furthermore, Fig. 6a shows that between T1 and R2, the first five helical paths do not intersect fouling A, which means that the amplitude of the wave packets should have no effect. This is confirmed by Fig. 8c, which shows that the difference between the two envelopes is negligible. A similar but mirrored effect is seen with fouling configuration B. With this configuration, the difference of signal envelopes measured between T1 and R1 remains negligible as shown in Fig. 8b, while Fig. 8d shows a noticeable drop in amplitude, in the predicted locations.

Based on these results, the two fouling cases can be distinguished from each other, and from the measurement on the clean pipe. In addition, this shows that the effect of fouling can be detected reliably in distinct wave packets, and thus, the location of the fouling phantom could be approximated.

**Figure 9:**
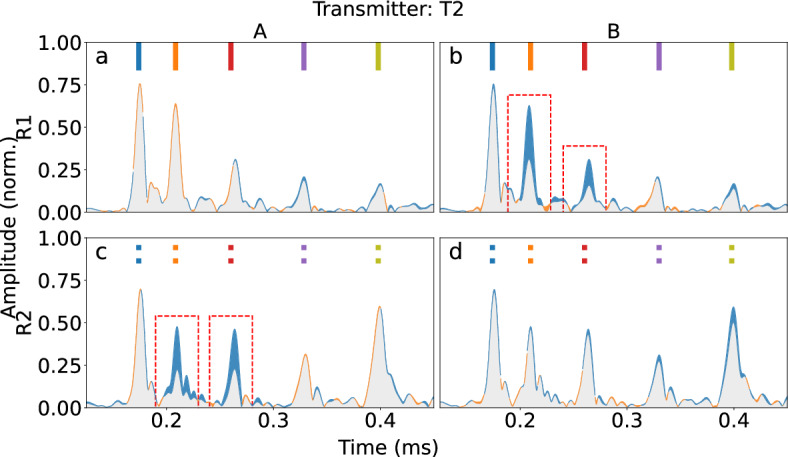
Envelopes of the time-domain signals where T2 is transmitting. The wave packets have been marked with bars following the color-coding of Fig. 6. The drops in amplitude (boxed) seen in this figure correspond to paths intersecting fouling in Fig. 6b.

The same analysis can be performed for the case where T2 acts as the actuator. In this case, the apparent amplitude drops should be seen in configurations (R1, B) and (R2, A), which is supported by the experimental results shown in Fig. 9. In this case, propagation paths 1 and 2 intersect with fouling when R2 is receiving in fouling configuration A (Fig. 9b), and while R1 is receiving in fouling configuration B (Fig. 9c). By combining the observations from all sensor pairs, the fouling proxy can be confidently localized.

In Figs. 8b,c and 9a,d, one can see that the last wave packet has a slight decrease in amplitude. This could be caused by scattering and reflection from the fouling phantom, even if it is not on any of the monitored propagation paths. In addition, the ray approximation is a simplification, while in reality the ultrasound source and pickup have finite dimensions.

## Discussion

The presence and rough location of the fouling can be inferred from the effect on the propagation paths seen by the transducer network. Fouling localization was intentionally demonstrated with a simple, pre-solved case. The goal of this work is to demonstrate that the sensor network can produce high-quality data to be used with a more sophisticated analysis method capable of solving arbitrary fouling cases.

The network was tested with custom-ordered transducers to ensure that the system is capable of fouling localization. Future work will include the manufacturing of self-made transducers to study the qualities, such as bandwidth and sensitivity, needed for the network to function. Preliminary testing shows that simple air-backed piezoelectric disks may be sufficient for fouling localization.

Localizing arbitrary fouling locations could potentially be possible with more sophisticated data analysis using the combined findings of the entire network. For example, machine learning methods could evaluate a multi-transducer network to localize and estimate the amount of fouling^35,36^. To reduce measurement time and power consumption, the model could interface directly with the sensors to leverage their real-time measurement capability and adjust the measurement interval of certain sensor subsets based on previously measured data. For example, the measurement interval could be increased in stable sections of a pipeline to reduce measurement time, while monitoring could be enhanced in an area with a sudden change in recorded signals.

Deploying the sensor network is easy, as it is wireless. Therefore, the sensors can be scattered flexibly on a structure or placed at regular positions depending on the use case. Flexible sensor placement also enables the use of algorithmic recommendations for optimal sensor locations for maximizing the information gain for a given task^36^. Regardless of configuration, evaluating the ultrasonic signals and determining the arrival times of the different propagation paths makes it possible to calibrate the exact locations after attaching the transducers. This simplifies the installation in industrial environments within confined spaces and difficult-to-reach installations. In addition, the RF transceiver used for triggering is often used for ranging the distance between two transceivers. By utilizing this capability in tandem with ultrasound time of flight measurements, one could map the network in a 3D space. This would further simplify the deployment of the sensors, as knowing the precise location of each sensor before measurements would not be necessary.

Even though we demonstrated the IoT platform in the specific task of localizing fouling, it is designed for general-purpose sensing and could directly be used for other ultrasonic applications. For example, the platform could be used to determine structures’ thickness by local pulse-echo measurements or monitor the growth of cracks, corrosion, or other defects. Additional instruments such as temperature sensors could be added for measurement or calibration purposes. The network can also be adapted to use other kinds of ultrasonic transducers such as magnetostrictive transducers^37^, electromagnetic acoustic transducers^38^, or contact-free laser excitation^39^.

## Conclusion

We present a cost-efficient and wireless IoT sensor platform that can detect fouling on pipe walls by monitoring ultrasonic signals. The main advantage of this hardware implementation is that the modules can be powered by batteries, which is especially desirable for low-repetition-rate measurements such as long-term monitoring of pipelines without readily available power supplies. Wireless configuration and data transfer further simplifies the integration of the platform into existing infrastructure and reduces the need for supporting hardware.

The sensor units work reliably and can detect changes in signals caused by fouling. Additionally, fouling localization was achieved by comparing signal amplitudes with the geometry of the setup. Localizing more complex fouling configurations could be possible with more advanced data analysis, e.g., using a machine learning model.

In summary, the presented IoT platform is a cost-efficient technology that enables continuous monitoring of industrial equipment on a large scale.

## Data Availability

Data recorded with the sensor network is available by reasonable request from the Electronics Research Laboratory (University of Helsinki, Finland).
